# Strategies for developing preparedness and building legacy – learning from the experience of building Health System Resilience in Ireland: Stephen Thomas

**DOI:** 10.1093/eurpub/ckac129.348

**Published:** 2022-10-25

**Authors:** S Thomas, P Fleming, C O'Donoghue, A Almirall-Sanchez

**Affiliations:** School of Medicine, Trinity College Dublin, Dublin, Ireland

## Abstract

Health system resilience to shocks is perhaps the biggest global challenge facing health systems in the 21st Century. Health systems face an increasing prevalence and likelihood of a broad range of shocks (including economic crises, pandemics, climate-related events, political upheavals, mass migration, conflicts and cyberterrorism) that can each undermine the ability of a health system to function well. In particular, the twin processes of dealing with the legacy of a health system shock and preparing for the next shock are distinct but related challenges that face policy makers today. In this presentation the authors will present key findings on improving preparedness and building a constructive legacy drawing from:

• the results of a recent systematic review on how health system resilience has been measured in high income countries over the last twenty years;

• the results of a recent realist review exploring the legacy of the economic crisis for the resilience of the response of the health system to COVID-19, and

• analysis of interviews with Irish policy makers, managers and analysts as they reflect on the different shocks encountered by the Irish system over the last fourteen years.

Triangulating these findings, the authors will reflect on the merits and challenges of measuring resilience and what the focus should be moving forward. Key strategies and approaches will be outlined to best prepare a system for a shock and to leave a positive legacy for the future.

